# 

**Published:** 1909-04-03

**Authors:** 


Telegraphic Address: Ospedale, London.

THE MODERN MEDICAL JOURNAL.

Telephone Number: 2734 Gerrard.

NEW SERIES. No. 109, Vol. V.
No. 1181. Vol. XLVI.

SATURDAY, APRIL 3RD, 1909.

PRICE THREEPENCE

